# Proposed Constitutive Law of Uniaxial Compression for Concrete under Deterioration Effects

**DOI:** 10.3390/ma13092048

**Published:** 2020-04-28

**Authors:** Yiwei Gao, Xuhua Ren, Jixun Zhang, Lingwei Zhong, Shuyang Yu, Xiang Yang, Wenbing Zhang

**Affiliations:** 1 College of Water Conservancy and Hydro-power Engineering, Hohai University, Nanjing 210098, China; gaoyiweijn@163.com (Y.G.); zhangjixun@hhu.edu.cn (J.Z.); zlwhhu@163.com (L.Z.); yushuyang_hhu@163.com (S.Y.); 15295571166@163.com (X.Y.); zwb0611@126.com (W.Z.); 2 Collaborative Innovation Center on Water Safety and Water Science, Hohai University, Nanjing 210098, China

**Keywords:** concrete, constitutive model, freeze–thaw cycles, ductile deformation

## Abstract

In order to study the ductile deformation characteristics and failure process of plain concrete under uniaxial compression, this paper proposes a new constitutive model. The new model was used to fit and analyze the constitutive curve of concrete under uniaxial compressive under various degradation forms and was compared with the traditional constitutive models. Finally, the new model was used to quantitatively analyze and predict the stress–strain curve of concrete in different degradation periods of a set of freeze–thaw measured data. The results show that, compared with the traditional constitutive model, the new model is simple in form and has few parameters, and the numerical value of the parameter can reflect the ductile deformation capacity of concrete. The fitting curve of the new model has the highest fitting degree with the measured stress–strain curve of concrete, and the goodness of fit (*R*^2^) is also the largest. The new model is suitable for fitting the stress–strain curve of concrete under uniaxial compression under various deteriorating forms, and the degree of fit between the constitutive prediction curve and the measured curve is high. It can be seen from the fitting results of the new model parameters that the ductile deformation capacity of concrete decreases first and then increases slightly, which is inconsistent with the law of gradual deterioration of strength. There is a minimum moment of ductility deformation capacity of concrete (MDC). The MDC of O-C40 concrete is about 114 freeze–thaw cycles, and the MDC of O-C50 concrete is about 116 freeze–thaw cycles; the degree of fit between the constitutive prediction curve and the measured curve is high. We hope that the improvement mentioned offers valid reference to the study of ductile deformation characteristics and failure process of compressed concrete under different deterioration forms.

## 1. Introduction

Concrete material is one of the most important materials in the world. It is known as being “food for construction industry”. For the past 40 to 50 years, at the early stage of engineering design, the initial investment cost of the project was often emphatically considered. However, the influence of engineering environment on concrete durability is ignored [1,2]. As a result, concrete buildings are damaged by freeze–thaw cycles, wet–dry cycles, salt ion erosion, alkali-aggregate reactions, and so on [3,4,5], which seriously affect the normal benefits and safe use of the structure. The process is gradually deteriorating from the surface to the inside, which is intuitively expressed as the mass loss and strength of the concrete [6,7]. Therefore, the mass loss rate, relative dynamic elastic modulus, and compressive strength have also become common indicators for studying the durability of concrete under different deterioration forms [8,9]. However, in the actual concrete structure, the stress state of concrete is complex and changeable, and the above indexes can only reflect the macroscopic mechanical properties of concrete, but it is difficult to fully reflect the mechanical properties and deformation characteristics of concrete.

The uniaxial compressive stress–strain curve of concrete is a comprehensive macroscopic reaction of the basic characteristics of compressed concrete [10], which can fully reflect the ductile deformation characteristics and failure process of concrete at every compressed stage. The uniaxial compressive stress–strain curve contains important mechanical performance index [11,12], such as the appearance and development of plastic strain of concrete, interior defects and damage, residual strength and deformation, etc. Through fitting analysis of the constitutive curve, the geometric shape of the curve and the mechanical properties of concrete can be quantitatively studied and predicted. The damage to the mechanical properties of concrete caused by various destructive effects will change the compactness, strength, and surface state of concrete and will inevitably cause the change of the shape of the uniaxial compression constitutive curve, so the fitting result will also change [13,14]. Strength and deformation properties of concrete under uniaxial compression determines characteristics of the overall mechanical performance of the concrete structure [15], which is also the main basis for studying the deformation and bearing capacity of concrete structures [16]. Therefore, it is of great significance to carry out research on the fitting of the uniaxial compression constitutive curve of concrete for practical engineering.

In the past few decades, some experts and scholars have proposed various constitutive models to analyze the uniaxial compression stress–strain curve of concrete [17,18]. These models can be divided into five categories: polynomial, exponential, trigonometric function, rational fraction, and piecewise. In the exponential constitutive model, y increases rapidly in the initial stage of the ascending curve and decreases too fast in the descending curve, so it was eliminated. The characteristics of trigonometric functions are the same as exponential one; after the descending curve intersects the x-axis, y becomes negative and the equation is invalidated, so it was also eliminated. At present, polynomial, rational fraction, and piecewise constitutive model are relatively commonly used. Reference [19] proposed the earliest polynomial constitutive model, which contains only one equation without parameters. This model can calculate the general outline of the measured stress–strain curve of concrete. Based on the model of [19], reference [20] proposed a piecewise constitutive model with the ascending segment in the form of polynomial and the descending segment in the form of a rational fraction. The ascending segment refers to the curve before the peak stress while the descending one refers to the curve after the peak stress. The model of [20] can calculate the general outline of the stress–strain curve, but the model contains no parameters, and it is difficult to ensure the accuracy of the fitting curve. In order to improve the fitting degree between the constitutive model and the measured stress–strain curve, reference [21] proposed a fourth-order polynomial equation and a rational fraction equation with a third-order polynomial denominator, respectively. Both models have a high degree of fit, but both models mention many parameters which have no clear physical meaning. Therefore, it is difficult to reflect the mechanical properties of concrete by parameter values. Based on the Weibull material strength statistical theory, reference [22] proposed a constitutive model applied for freeze–thaw concrete. The parameters of this model have clear physical meanings, but the deviation of the descending curve from the measured results is large. Reference [23] proposed a constitutive model for plain concrete in compression, and the form of the model is simple. The parameters that define the relationship are physically significant and can be estimated from empirical relationships or determined experimentally. However, the maximum stress is determined in accordance with ASTM C 39, “Standard Test Method for Compressive Strength of Cylindrical Specimens”. Therefore, this model is mostly used in cylindrical specimens, but less in other specimens. In order to improve the fitting degree of the measured curve and to reflect the mechanical properties of concrete by the parameter values, reference [17] proposed a piecewise constitutive model with two segments. The ascending one is in the form of a third-order parabola, while the descending one is in the form of a rational fraction [24]. The model is simple in form and has few parameters with clear physical meaning. The model of [17] is commonly used at present [25,26] on account of the good fitting degree. However, the fitting degree of the model of [17] is still unsatisfactory when the concrete deterioration is critical [25]. The research results noted above enrich the study on constitutive model of concrete under uniaxial compression. However, due to the complexity of service environment of concrete buildings, the process of concrete deterioration is complex and changeable. In order to describe the plastic deformation process and failure process of concrete under compression more reasonably, it is necessary to carry out further research on the constitutive model of concrete under uniaxial compression. 

Based on the previous research results, this paper finds that the piecewise constitutive model is easier to accurately fit constitutive curve of uniaxial compression, the polynomial equation is suitable for fitting the ascending curve, and the rational fraction equation is suitable for fitting the descending curve. In view of this, based on the model of [17], this paper proposes an equation in the form of a fourth-order polynomial in the ascending section. According to the model constraint conditions proposed by [17], the constraint calculation of this equation is carried out in order to make it contain single model parameters with clear physical meaning, the descending curve is still using the rational fraction equation proposed by [17]. First, based on the previous measured results of [27], we used the new model and traditional constitutive models to analyze the measured stress–strain curve and compare their respective fitting characteristics and advantages. Then, the new model was used to fit and analyze the constitutive curve of concrete under uniaxial compressive under various degradation forms, to verify its applicability [25,28,29,30]. Finally, the new model was used to quantitatively analyze and predict the stress–strain curve of concrete in different degradation periods of the measured freeze–thaw data of [27]. This paper hopes to provide a theoretical basis for the performance analysis and stability prediction of concrete structures in different deterioration environments.

## 2. Seven Constitutive Models

### 2.1. The Hognestad et al. (1955) Model

Reference [19] proposed a unified polynomial constitutive model for the ascending curve and the descending curve, as shown in Equation (1). This model is dimensionless and has few parameters, simple form, and convenient calculation. Before this model was proposed, there were few studies on constitutive model; the proposal of this equation has great guiding significance for the development of the constitutive model later. The similarity degree of the measured curve and the calculation curve can be observed intuitively by putting them in a graphical form:

(1) $$f=2\varepsilon -\varepsilon^{2}$$

where *f* is the stress in the constitutive model of concrete under uniaxial compression, and *ε* is strain.

### 2.2. The Rüsch et al. (1955) Model

In order to meet the actual needs of the project, reference [20] carried out uniaxial compression tests of concrete under different loading rates and improved Equation (1). The complex continuous stress–strain curve is simplified into ascending section, which is in the form of the parabolic and descending section that is in the form of a straight line. This equation is a dimensionless equation in simple form; it is favored by engineers and has been incorporated into the CEB-FIP MC90 specification [31]. This is the earliest piecewise constitutive equation, after which the piecewise constitutive model has a great development. For example, the piecewise constitutive equation proposed by [17] is highly recognized at present. Using the same method demonstrated in Section 2.1, it is possible to observe the similarity degree between the calculation curve and the fitting curve.

However, there are some differences between the fitting results of this equation and the measured curves; for instance, the peak of the curve is discontinuous, and the descending section curves of different concrete are very different.

(2) $$f=\{\begin{array}{c} 2\varepsilon -\varepsilon^{2}\begin{array}{cc} & 0\leq \varepsilon \leq \varepsilon_{pr} \end{array}\\ 1-0.15\left(\frac{\varepsilon -1}{\varepsilon_{u}-1}\right)\varepsilon \geq \varepsilon_{pr} \end{array}$$

In Equation (2), *ε_pr_* is the peak strain, and *ε_u_* is the strain corresponding to 0.85*f_pr_* (peak stress) in the falling section. 

### 2.3. The Saenz et al. (1964) Model

In order to improve the fitting ability of the stress–strain full curve of concrete, reference [21] used the fourth-order equation to analyze the constitutive curve; as a result, the model parameters were increased, and the equation form was a dimensionless polynomial, as shown in Equation (3). By fitting the measured curves, the values of parameters *c*_1_, *c*_2_, *c*_3_, and *c*_4_ can be obtained. Substituting parameters mentioned above into Equation (3) can get the fitting curve of Equation (3). The fitting degree of the measured curve and the fitting curve can be observed intuitively by putting them in a graph, or by the goodness of fit (*R*^2^).

(3) $$f=c_{1}\varepsilon +c_{2}\varepsilon^{2}+c_{3}\varepsilon^{3}+c_{4}\varepsilon^{4}$$

### 2.4. The Saenz et al. (1964) Model

When [21] proposed Equation (3), in order to increase the diversity of fitting results of concrete constitutive curve; they proposed a rational fraction dimensionless constitutive model with the denominator of third order, and the model contains 4 parameters, as shown in Equation (4). Using the same method as in Section 2.3, the fitting degree between the measured curve and the fitting curve can be observed.

(4) $$f=\frac{\varepsilon }{c_{1}+c_{2}\varepsilon +c_{3}\varepsilon^{2}+c_{4}\varepsilon^{3}}$$

### 2.5. The Guan et al. (2014) Model

In order to introduce the damage variable into the constitutive model to characterize the degradation of concrete materials, reference [22] established a constitutive model of freeze–thaw damage of concrete based on Weibull material strength statistical theory and meso-statistical damage mechanics, and according to the uniaxial compression test of freeze–thaw concrete, the parameter *m* of the model is fitted. Through the experimentation, reference [22] proved the model can accurately fit the uniaxial compression constitutive curve of concrete materials under freeze–thaw cycles, and also can describe the damage evolvement process of concrete. The model of [22] is an emerging constitutive model mainly used in the field of freeze–thaw concrete, as shown in Equation (5). The similarity degree of the calculation curve and the fitting curve can be observed intuitively by putting them in a graph.

(5) $$f=E\varepsilon \exp\left[-\frac{1}{m}{\left(\frac{\varepsilon }{\varepsilon_{pr}}\right)}^{m}\right]$$

In Equation (5), *m* = *1/(ln(Eε_pr_/f_pr_))* is a non-negative number; *E* is the initial elastic modulus of concrete; and *ε_pr_* and *f_pr_* represent peak strain and peak stress, respectively. 

### 2.6. The Carreira and Chu (1985) Model

Reference [23] proposed a constitutive model for plain concrete in compression, which is suitable for the constitutive relation of cylindrical specimens. As shown in Equation (6), the model contains an equation, which can be used to fit the ascending and descending sections of the stress–strain curve, respectively. Reference [23] obtained the parameters of the model through empirical relationships or determined them experimentally. Reference [32] obtained the parameters of the model through numerical calculation. This paper adopts the method in Reference [32]; due to the complexity of the calculation process of the model parameters in Reference [32], there is no need to enumerate them here.

(6) $$f=f_{pr}^{\prime }\frac{\beta \left(\varepsilon /\varepsilon_{pr}^{\prime }\right)}{\beta -1+{\left(\varepsilon /\varepsilon_{pr}^{\prime }\right)}^{\beta }}\begin{array}{c} \beta =\beta_{1}\\ \beta =\beta_{2} \end{array}\begin{array}{cc} & 0\leq \varepsilon \leq \varepsilon_{pr}^{\prime }\\ & \varepsilon >\varepsilon_{pr}^{\prime } \end{array}$$

In Equation (6), *β*_1_ and *β*_2_ are material parameter that depends on the shape of the stress–strain curve. Reference [32] points out that parameters *β*_1_ and *β*_2_ can be obtained by calculation. When 0 ≤ *ε* ≤ *ε_pr_*, by substituting the value of parameter *β*_1_ into Equation (5), the fitting result of the rising curve can be obtained, and the value of parameter *β*_2_ adopts the same method can get the descent curve when *ε_pr_* ≤ *ε*. *f_pr_*′ is the maximum stress, usually considered as the concrete strength and usually determined in accordance with ASTM C 39, “Standard Test Method for Compressive Strength of Cylindrical Specimens”. Moreover, *ε_pr_*′ is the strain corresponding with *f_pr_*′. Since the prism specimen is adopted in this paper, in order to ensure that the peak stress and the peak strain of the fitting curve are equal to the values of the measured curve, the peak stress *f_pr_* is used to replace *f_pr_*′ and the peak strain *ε_pr_* to replace *ε_pr_*′.

### 2.7. The Guo et al. (1997) Model

Reference [17] proposed a piecewise constitutive model, which contains two equations and each equation contains only one parameter. The first equation is in the form of a third order parabola and the second equation is in the form of a rational fraction. The first equation can obtain the value of parameter *a* after fitting the ascending segment of the stress–strain curve of concrete (the curve before the peak stress). The second equation can obtain the value of parameter *b* after fitting the descending segment of the stress–strain curve of concrete (the curve after the peak stress). By substituting the value of parameter *a* into the first equation, the fitting result of the rising curve can be obtained, and the value of parameter *b* adopts the same method. This constitutive model is widely used at present, as shown in Equation (7).

(7) $$\{\begin{array}{ll} y=ax+(3-2a)x^{2}+(a-2)x^{3} & \begin{array}{cc} & 0\leq x\leq 1 \end{array}\\ y=\frac{x}{b{\left(x-1\right)}^{2}+x} & \begin{array}{cc} x>1 & \end{array} \end{array}$$

In Equation (7), *y* = *f/f_pr_*, *x* = *ε/ε_pr_*. Parameters *a* and *b* have clear physical and geometric meaning [17]: Under normal circumstances, the parameter *a* value range from 1.5 to 3 and the parameter *b* values range from 0 to positive infinity, while the parameter *a* value may also be less than 1.5 or even negative. If *a* is smaller or *b* is larger, such as *a* is 0 and *b* is 10, the curve will be steeper, and the surrounding area under the curve will be smaller, indicating that the plastic deformation capacity and ductility deformation capacity of the concrete will be smaller, the material will be brittle, the failure process will be rapid, and the residual strength will be lower. On the contrary, the damage of concrete is slow and the residual strength is high. Therefore, the parameters *a* and *b* can be used to compare or measure the differences in the mechanical properties of concrete.

**Figure 1:**
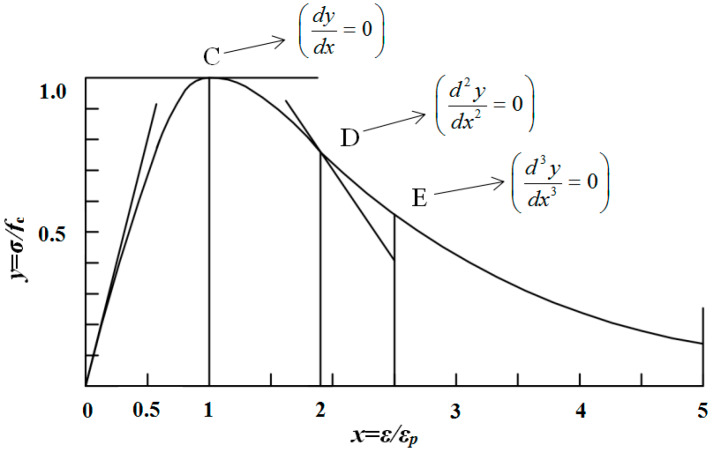
Professor Guo’s suggested equation curve.

The theoretical description of the full curve of [17] is shown in Figure 1; dimensionless coordinates are used in the drawing. 

(1) *x* = 0, *y* = 0;

(2) 0 ≤ *x* < 1, the slope of the curve decreases monotonically, and the curve has no inflection point;

(3) *x* = 1, *y* = 1, *dy/dx* = 0, the curve is a single value peak;

(4) *x* > 1, the curve has an inflection point D;

(5) *x* > 1, the descending curve has the maximum curvature point E;

(6) *x*→ ∞, y→ 0, *dy/dx*→ 0; 

(7) *x* ≥ 0, 0 ≤ *y* ≤ 1.

### 2.8. The New Model

At present, most of the constitutive models are unified full curve equations for the ascending curve and the descending curve. However, it is difficult to choose a single curve equation with simple form which can satisfy the geometric conditions of the constitutive curve and adjust the exact shape of the curve according to different concrete, so the form of the new constitutive model is piecewise. Reference [17] proposed that the curve of ascending segment in the form of a third-order parabola has a higher fitting degree to the constitutive curve of concrete with better mechanical properties, but it has a low fitting degree to the constitutive curve of concrete, with poor mechanical properties [25]; the descending curve of rational fraction has a good fitting degree to the constitutive curve of concrete with various mechanical properties; and both the curve equation of the ascending section and the curve equation of the descending section contain model parameters with clear physical meaning, which can be used to describe the mechanical properties of concrete. Thus, the descending curve equation follows the rational fraction equation proposed by [17]. The ascending curve equation adopts Equation (3), which is one order higher than Equation (7). The parametric equation can be obtained by combining with the three boundary conditions of Features (1), (2), and (3) (*x* = 0, *y* = 0; *x* = 1, *y* = 1; *x* = 1, *dy/dx = 0).*

Because Equation (3) contains four parameters, in order to get a single parametric equation, one of the four parameters needs to be zero; when *c*_4_ is zero, the equation has the same form as Equation (7) when *c*_1_, *c*_2_, and *c*_3_ are zero. The equations are shown in Equations (8) to (10):

(8) $$y=c_{2}x^{2}+c_{3}x^{3}+c_{4}x^{4}$$

(9) $$y=c_{1}x+c_{3}x^{3}+c_{4}x^{4}$$

(10) $$y=c_{1}x+c_{2}x^{2}+c_{4}x^{4}$$

In Equations (8)–(10), *y* = *f/f_pr_*, *x* = *ε/ε_pr_*. Combing with *x* = 0, *y* = 0; *x* = 1, *y* = 1; *x* = 1, *dy/dx* = 0, Equations (8)–(10) can be transformed into Equations (11)–(13):

(11) $$y=\left(3+c\right)x^{2}-\left(2+2c\right)x^{3}+cx^{4}$$

(12) $$y=\left(1.5+0.5c\right)x-\left(0.5+1.5c\right)x^{3}+cx^{4}$$

(13) $$y=\left(2+2c\right)x-\left(1+3c\right)x^{2}+cx^{4}$$

Taking Equation (12) as an example, when *x* = 0, *dy/dx* = 1.5 + 0.5*c*, we get the following:

(14) $$1.5+0.5c={\frac{dy}{dx}|}_{x=0}={\frac{df/f_{pr}}{d\varepsilon /\varepsilon_{pr}}|}_{x=0}=\frac{{df/d\varepsilon |}_{x=0}}{f_{pr}/\varepsilon_{pr}}=\frac{E_{O}}{E_{P}}$$

In Equation (14)*, E_O_* = *d_f_ /d_ε_|_x=0_* is the initial tangent elastic modulus of concrete, N/mm^2^; and *E_P_* = *f_pr_/ε_pr_* is the peak secant modulus of concrete, N/mm^2^.

Equation (15) can be obtained from Equation (14):

(15) $$c=2\times \frac{E_{O}}{E_{P}}-3$$

It can be seen that *c* has a clear physical (geometric) meaning—the ratio of the initial tangent modulus to the secant modulus times 2 minus 3. 

By condition (2), when 0 ≤ *x* ≤ 1, *d^2^y/dx*^2^ = −6(0.5 + 1.5*c*)*x* + 12*cx*^2^ < 0, the available range of *c* is as follows:

(16) $$-\frac{1}{3}\leq c\leq 1$$

**Figure 2:**
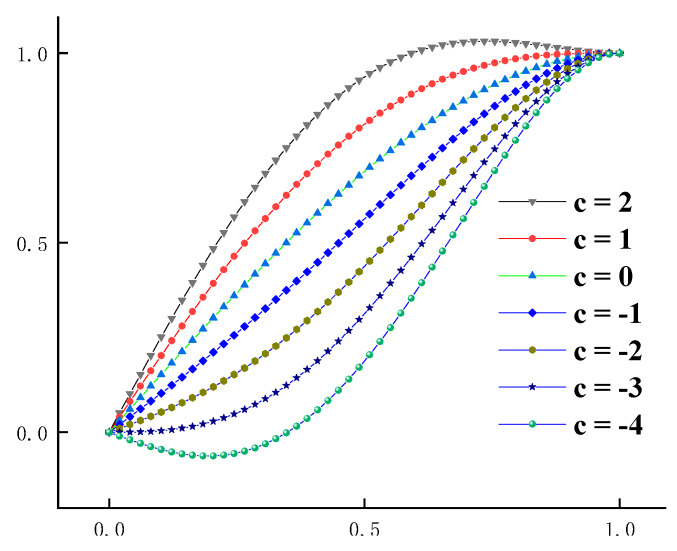
Dimensionless fitting results of the new model of stress–strain curve of concrete under uniaxial compression.

By fitting the ascending section of the stress–strain curve based on Equation (12), and then getting the value of the parameter *c*, the fitting curve of Equation (12) can be obtained by substituting the fitting result of *c* into the equation. The corresponding relationship between the different value of parameter *c* and the curve shape before the peak stress is shown in Figure 2. If *c* > 1, y > 1.0, in the local area of the rising curve, part of the stress is higher than the peak stress before the peak point, which obviously violates the actual situation. When −1/3 < *c* < 1, there is an inflection point on the curve, but the inflection point and the curvature are not obvious. When *c* < −1/3, with the decrease of *c*, the curvature of the curve increases gradually, and a large deformation will occur under small stress. The shape of the uniaxial compression curve of concrete can reflect the mechanical properties of concrete, so there is a certain relationship between the value of parameter *c* and the mechanical properties of concrete. 

In Section 2.7, the fitting equation of the curve of the stress–strain ascending section contains only one parameter *a*, and the value of parameter *a* can reflect the ductile deformation capacity of concrete. Equation (12) is derived from Equation (7) and it contains only one parameter *c*, so it can be known from Section 2.7 and Figure 2 that *c* has a clear physical meaning: If *c* is smaller, the curve will be steeper and the surrounding area under the curve will be smaller, the plastic deformation capacity and ductility deformation capacity of the concrete will be smaller, the failure process will be rapid, and the residual strength will be lower. In contrast, the ductility deformation capacity of concrete and residual strength will be higher. Therefore, the mechanical properties of concrete can be measured by the numerical value of parameter *c*.

By using the same method, it can be concluded that the parameter *c* in Equation (11) is meaningless. Using Equation (11) to fit the ascending section of the stress–strain curve can get the value of the parameter *c*. The mechanical properties of concrete represented by parameter *c* are the same as Equation (12); the range of *c* is as follows:

(17) −3≤c≤0

It can be seen that *c* has a clear physical (geometric) meaning—the ratio of the initial tangent modulus to the secant modulus is reduced by 2 and then multiplied by −0.5. Using Equation (13) to fit the ascending section of the stress–strain curve can get the value of parameter *c*. The mechanical properties of concrete represented by parameter *c* are the same as Equation (12); the range of *c* is as follows:

(18) $$-\frac{1}{3}\leq c\leq \frac{1}{3}$$

## 3. Method and Materials

### 3.1. Concrete Mix Proportion

Reference [27] designed four groups of concrete specimens with the strength grade of concrete and the amount of air entraining agent as variables, respectively numbered as Ordinary C40, Ordinary C50, Air-entraining C40, and Air-entraining C50; these four groups of concrete are respectively referred to as O-C40, O-C50, AE-C40, and AE-C50. The concrete mix proportion is shown in Table 1.

### 3.2. Measured Results

**Figure 3:**
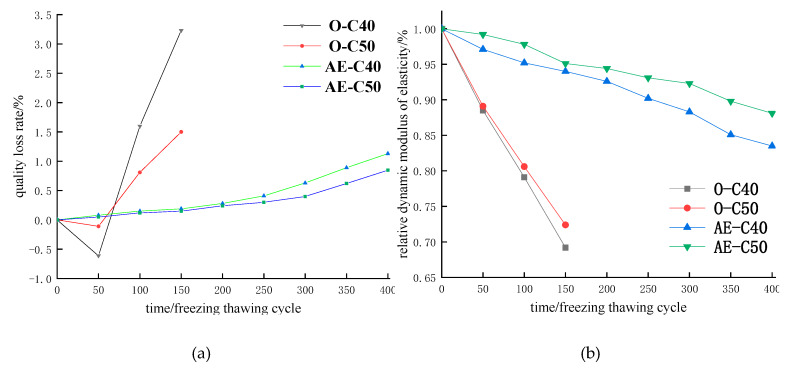
Mass loss rate and relative dynamic elastic modulus of concrete after freeze–thaw cycles: (**a**) quality loss rate of concrete; (**b**) relative dynamic modulus of elasticity of concrete.

**Figure 4:**
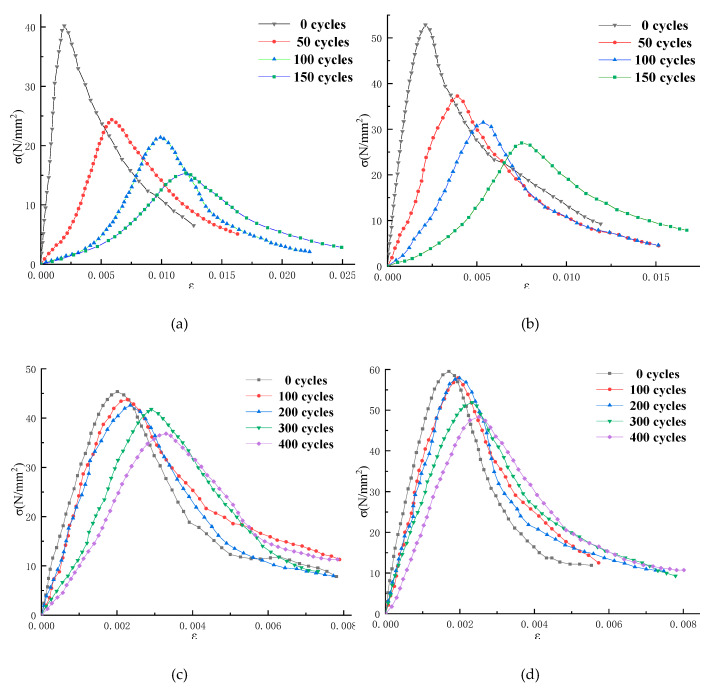
Stress–strain curve of concrete specimens under uniaxial compression after freeze–thaw cycles: (**a**) O-C40 concrete; (**b**) O-C50 concrete; (**c**) AE-C40 concrete; and (**d**) AE-C50 concrete.

The concrete specimens are cast in a size of 100 mm × 100 mm × 300 mm, and the freeze–thaw test is performed according to the “Test method for long term performance and durability of ordinary concrete” (GB50082-2009). Each freezing-and-thawing cycle is completed within 2 to 4 h; the melting time is more than 1/4 of the time of a freezing-and-thawing cycle. The central temperature of concrete specimens at the end of freezing and thawing is −18 ± 2 °C and 5 ± 2 °C, respectively. Dynamic modulus of elasticity and mass of concrete specimens are measured every 50 freezing-and-thawing cycles, as shown in Figure 3. Concrete uniaxial compression test is carried out on a WAW-1000 microcomputer-controlled electro-hydraulic servo universal testing machine, using a displacement-controlled constant loading rate method with a loading rate of 0.1 mm/min. For ordinary concrete, the stress–strain curve is tested when the number of freezing and thawing cycle is 0, 50, 100, and 150; and the stress–strain curve of air-entrained concrete is tested when the number of freezing-and-thawing cycle is 0, 100, 200, 300, and 400, as shown in Figure 4.

## 4. Result and Discussion

### 4.1. Comparison of Fitting Results of Constitutive Models

**Figure 5:**
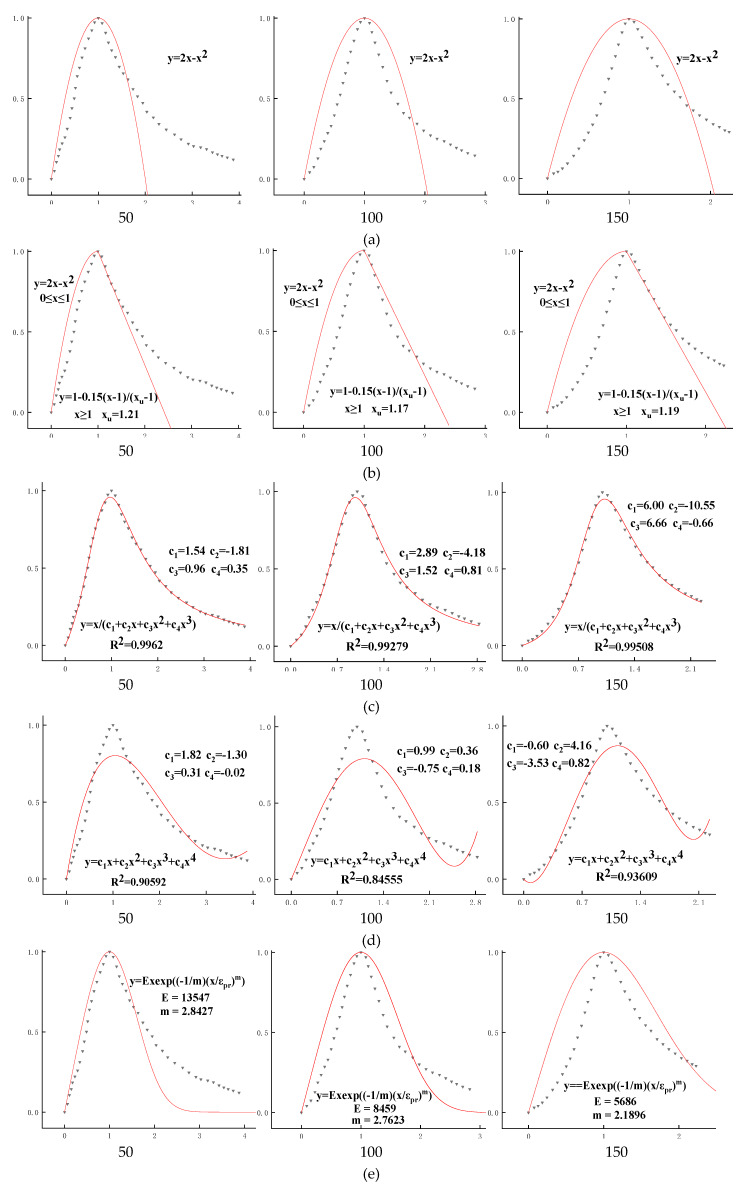

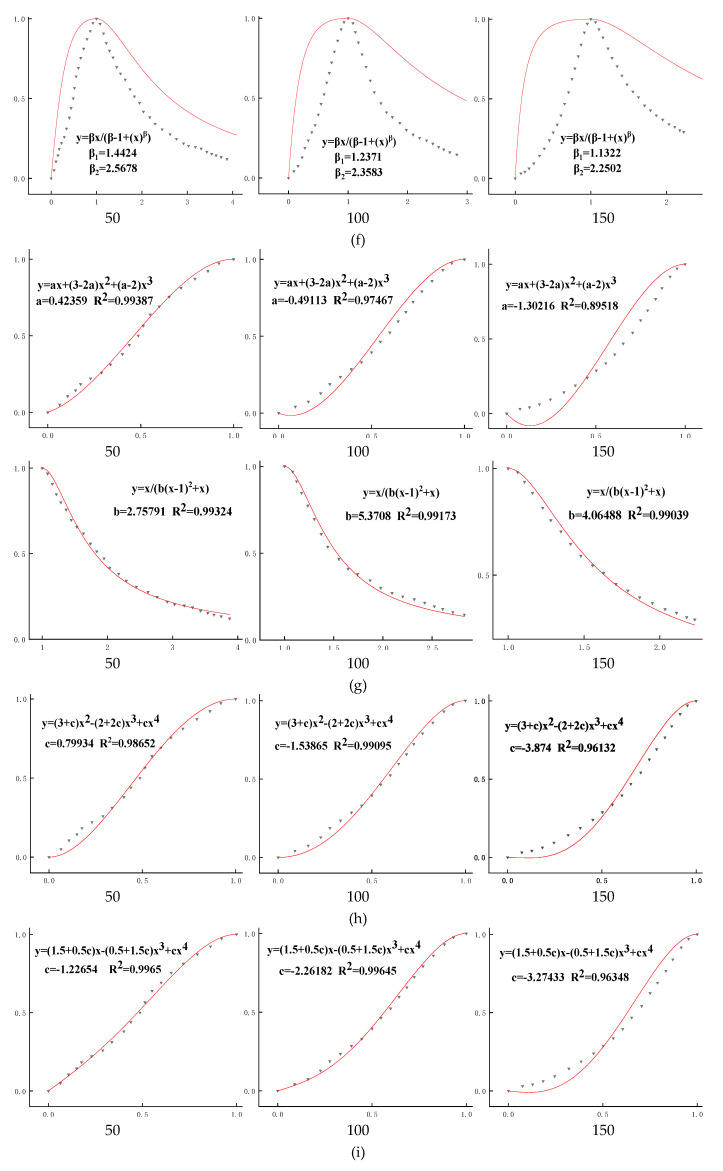

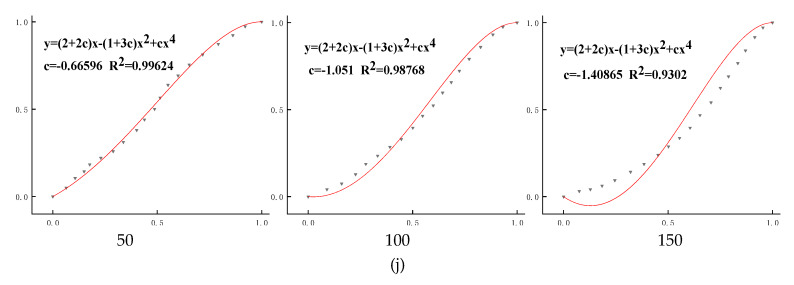
Fitting results of constitutive model of freeze–thaw concrete: (**a**) reference [19]; (b) reference [20]; (c) reference [21]; (d) reference [21]; (e) reference [22]; (f) reference [23]; (g) reference [17]; (**h**) the new model one; (**i**) the new model two; (**j**) the new model three; 50, 100, and 150 are numbers of freeze–thaw cycles.

Put the measured curve and the calculated curve or fitting curve in the same graph, so that their fitting degree can be observed. In order to prevent the accidental results of the fitting results, 50, 100, and 150 cycles of freeze–thaw cycles were conducted on ordinary C50 concrete. The results of stress–strain curves are shown in Figure 5. The black triangle dot is the measured result, and the red line is the fitting result; dimensionless coordinates (*x = ε/ε_pr_*, *y = f/f_pr_*, *x_u_ = ε_u_/ε_pr_*) are used in the figure. Figure 5a–g shows the fitting results of Equations (1) to (7), respectively, and Figure 5h–j shows the fitting results of Equations (11) to (13), respectively. Among them, the graphs of Figure 5a–f are the fitting results of the complete stress–strain curve for concrete; Figure 5g contains the fitting results of the ascending section and the descending section of the stress–strain curve; and Figure 5h–j shows the fitting results of the ascending section of the stress–strain curve for concrete.

Figure 5 shows the fitting results of seven traditional constitutive models and three new constitutive models. It can be seen that the model of [19], although simple in form and easy to calculate, has a large difference between the measured curve and the fitting result, and the fitting result is difficult to reflect the process of failure deformation and damage accumulation after the concrete is compressed. The fitting result of the ascending curve of the model of [20] is the same as that of the model of [19], which is different from the measured curve. This model can hardly reflect the change of residual stress and the plastic deformation process of concrete. Reference [21] (Equation (3)) has too many parameters, and the parameter changes have no regularity to follow, so it is difficult to describe the change of the mechanical properties of concrete through the parameter values. The goodness of fit (*R*^2^) is less than 0.94; the fitting results are quite different from the measured curve, which is also difficult to reflect the deformation and failure process of concrete under compression. The fitting results of [21] (Equation (4)) have a high integration degree with the measured curve, and the goodness-of-fit results are all above 0.99, which can well describe the geometrical shapes of the stress–strain curve of concrete. However, there are too many parameters in the model, and the change of parameters are irregular; it is also difficult to describe the mechanical properties of concrete through the value of the parameters. The model parameters in [22] are few and have clear physical significance, which can reflect the mechanical properties of concrete, but there are some differences between the fitting result and the calculated curve, so the mechanical properties of the concrete obtained through the parameter values are not accurate. The calculated curve of the model of [23] is quite different from the measured curve. Therefore, it is difficult for this model to fit the shape of the stress–strain curve of concrete. Since the prism specimen is used in this paper, and the model of [23] is applicable to the cylinder specimen. Therefore, there is some difference between the calculated curve and the measured curve.

Both the ascending curve equation and descending curve equation of the model of [17] have only one parameter, which is easy to calculate; the parameters are independent of each other and have clear physical meaning, so they can fit the ascending curve and the descending curve. When the freeze–thaw cycle is 50 times, the measured results of the ascending section of the concrete constitutive curve has a high integrating degree with the fitting results, and goodness of fit is above 0.99. However, with the deepening of the deterioration of concrete, the difference between measured curve of the ascending section and the fitting result increases gradually; when the freeze–thaw cycle is 150 times, the goodness of fit is below 0.90, but it can still roughly describe the outline of the ascending section curve. The measured results of the descending section of the constitutive curve has a high integrating degree with the fitting results; the goodness of fit are all above 0.99, which can accurately reflect the plastic deformation process of concrete and the change of residual stress. The fitting results of the three new models have a high integration degree with the measured results; by comparing the goodness of fit, it can be found that the *R*^2^ of the new model two is the highest and it also has the best fitting result. When the freeze–thaw cycle is 50 and 100 times, *R*^2^ is as high as 0.9965 and 0.99645. Respectively, the fitting results are better than any one of the constitutive models. when the freeze–thaw cycle is 150 times, the deterioration of concrete is more serious, but the goodness of fit of the new model two is more than 0.96, indicating that the new model two can be used to “precisely” fit the rising section of the uniaxial compression constitutive curve of freeze–thaw concrete. 

**Figure 6:**
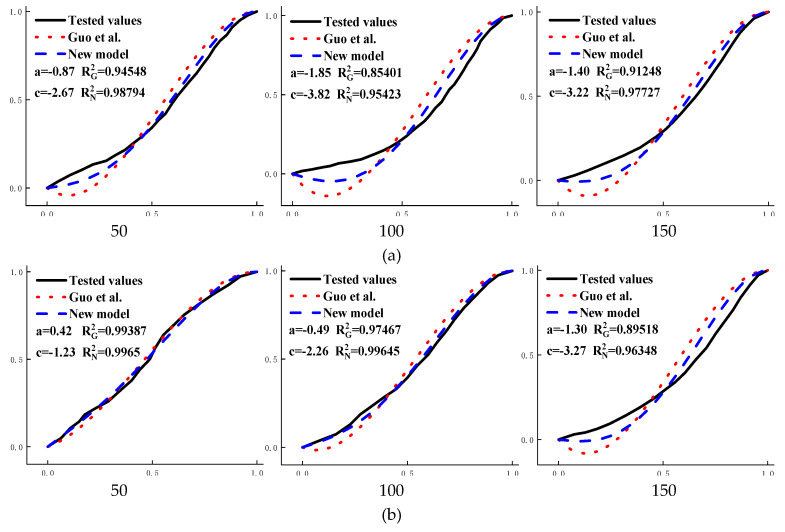
Comparison results between the model of [17] and the new model two: (**a**) O-C40 concrete; (**b**) O-C50 concrete; 50, 100, and 150 are numbers of freeze–thaw cycles.

By comparison, it is found that the fitting results of the model of [17] and the new model two have a high integration degree with the measured curve, at present, the fitting of constitutive curve of concrete under uniaxial compression is more accurate and popular by [17]. Figure 5g shows that the fitting results of the descending curve fitted by the model of [17] are relatively accurate, but under the same freeze–thaw times, the goodness of fit of the ascending curve section of the model of [17] is less than that of the new model two. In order to compare the accuracy of the fitting results of the two models more reasonably, the stress–strain curve of 50, 100, and 150 times of freezing and thawing of ordinary C40 concrete (Figure 6a) and ordinary C50 concrete (Figure 6b) were selected, and the fitting results of the two models were compared in one picture, as shown in Figure 6.

It can be seen from Figure 6 that the ordinary C50 concrete with 50 times of freeze–thaw cycle has higher strength, the fitted results of the two models are almost seamlessly connected with the measured curve, and *R*^2^ results are all above 0.99. As the deterioration of the concrete deepens, the degree of deviation between the fitting result fitted by the model of [17] and the measured curve is gradually increase. When the freeze–thaw cycle was 150 times, *R^2^_G_* (the goodness of fit of the model of [17]) was less than 0.9. The fitting curve of the model of [17] decreases first and then increases, meaning that the stress before the curve rises is less than zero. Therefore, it is obviously not in line with the actual law. The fitting result of the new model two gradually deviates from the measured curve with the deepening of concrete deterioration, but *R^2^_N_* (the goodness of fit of the new model) is always higher than 0.96, and the fitting results always have a high integration degree with the measured curve. The fitting curve of the new model two is always surrounded by the fitting curve of [17]. In the ordinary C40 concrete with low strength, the fitting curve of [17] has negative stress areas. The maximum value of *R^2^_G_* is only 0.94, and the minimum value is as low as 0.85, so the fitted result has severely deviated from the measured curve; the minimum value of *R^2^_N_* of the new model two is 0.95, which is higher than the maximum value of *R^2^_G_* and always keeps a high integration degree with the measured curve. There is almost no negative stress area in the fitting results, which is more in line with the reality. In summary, the new model two proposed in this paper has a high integration degree to the measured curve of concrete, and almost no negative stress areas appear and is more in line with actual.

### 4.2. Applicability of the New Model

**Figure 7:**
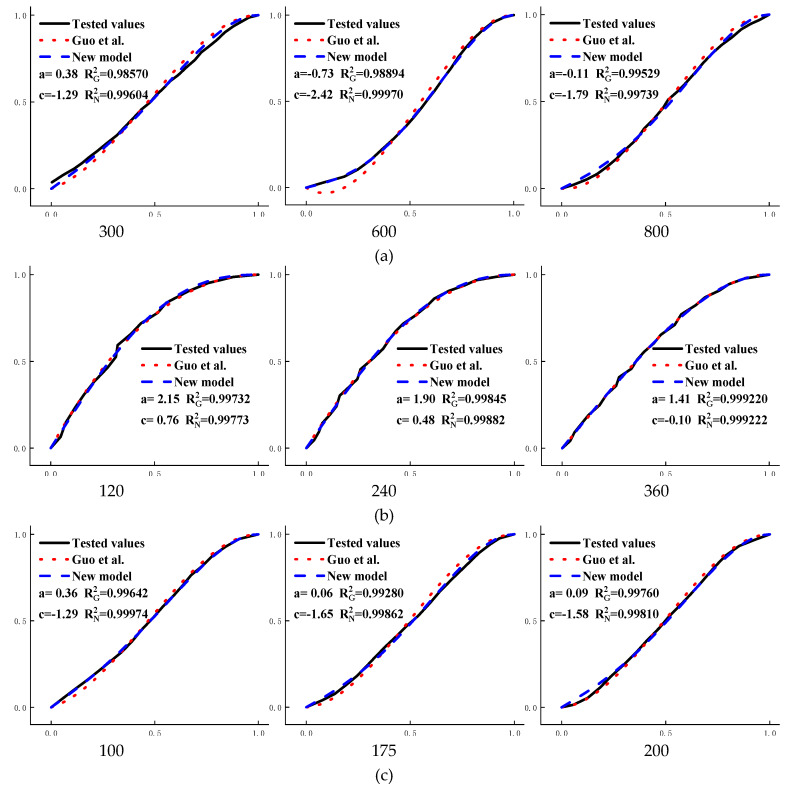

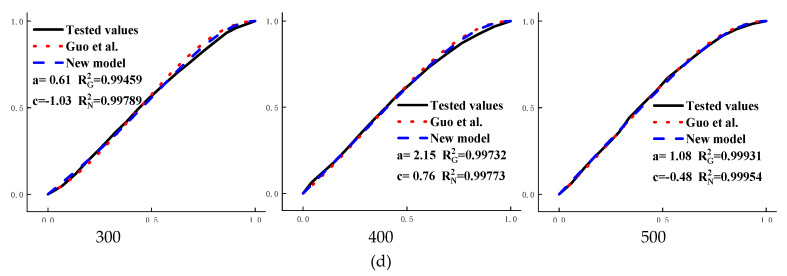
Comparison results between the model of [17] and the new model two: (**a**) high temperature failure of concrete, 300, 600, and 800 are exposure temperatures; (**b**) dry–wet cycle failure of concrete, 120, 240, and 360 are erosion days; (**c**) freeze–thaw cycle failure of concrete, 100, 175, and 200 are freezing-and-thawing times; (**d**) high temperature failure after 25 freeze–thaw cycles of concrete, 300, 400, and 500 are exposure temperatures.

In order to explore the applicability of the new model to the stress–strain curve of concrete under uniaxial compression under other state of deterioration, four groups of measured data of concrete under different deterioration situations were selected, which are high temperature failure, dry–wet cycle failure, freeze–thaw cycle failure, and high temperature failure after freeze–thaw cycle [25,28,29,30]. The fitting results of the model of [17] and the new model were compared in Figure 7.

The fitting curve of the new model has a high degree of integration with the measured curve. The goodness of fit of the new model is greater than that of the model of [17] and the fitting curve of the new model are surrounded by the fitting curve of the model of [17]. Therefore, the fitting results of the new model is the best.

Therefore, this paper uses Equation (19) to fit and analyze the measured stress–strain curve in Figure 4:

(19) $$\{\begin{array}{ll} y=\left(1.5+0.5c\right)x-\left(0.5+1.5c\right)x^{3}+cx^{4} & \begin{array}{cc} & 0\leq x\leq 1 \end{array}\\ y=\frac{x}{b{\left(x-1\right)}^{2}+x} & \begin{array}{cc} x>1 & \end{array} \end{array}$$

Here, *c* is the parameter in the fitting equation of the ascending curve (the curve before the peak stress), which can be obtained by fitting the curve before the peak stress; and *b* is the parameter in the fitting equation of the descending curve (the curve after the peak stress), which can be obtained by fitting the curve after the peak stress.

### 4.3. Fitting and Prediction of Stress–Strain Curve of Concrete

The stress–strain curve of concrete under uniaxial compression is the most basic constitutive relation, which can fully reflect the strength, toughness, and ductile deformation of concrete materials, etc. Equation (19) is used to fit the constitutive curve of concrete under uniaxial compression; the value of the parameter can be used to reflect the mechanical properties of concrete by fitting the value of parameters, and can also study the evolution law of mechanical properties of concrete through the change of parameters. The fitting parameters *c* and *b* of the measured curve in Figure 4 are shown in Table 2.

**Figure 8:**
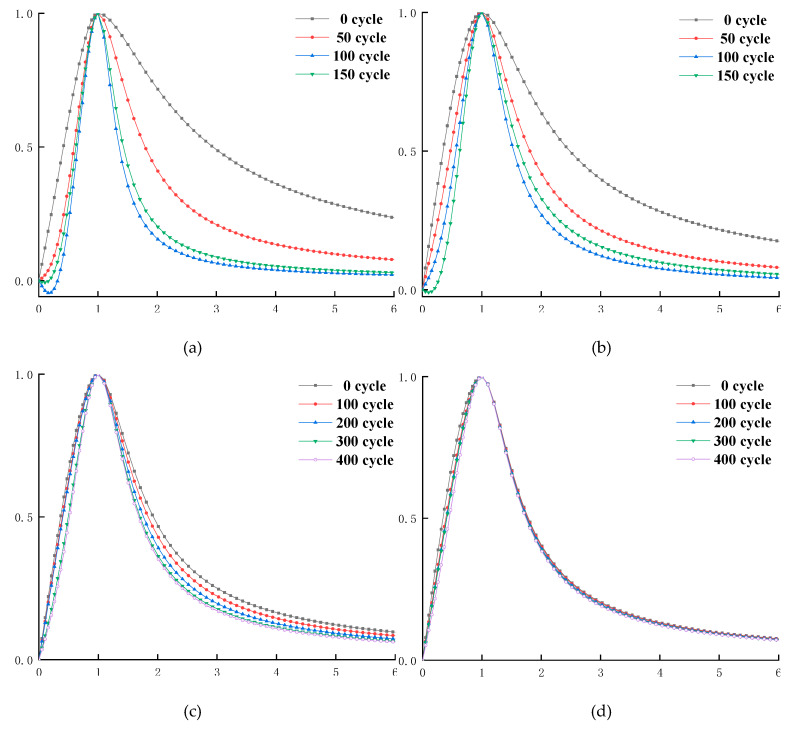
Dimensional fitting curve of stress–strain for freeze–thaw concrete under uniaxial compression: (**a**) O-C40 concrete; (**b**) O-C40 concrete; (**c**) AE-C40 concrete; and (**d**) AE-C40 concrete.

As can be seen from Table 2, with the increase of the number of freeze–thaw cycles, parameter *c* of AE-concrete is gradually decreased, parameter *b* is gradually increased, the curve becomes increasingly steep (Figure 8), and the lower surrounding area becomes increasingly small. From the geometric definitions of Section 2.7 and Section 2.8, we can know that the materials of AE-concrete become brittle gradually, ductility and residual strength become increasingly low, and the failure process is increasingly rapid; this is consistent with the law of the gradual decrease of the mechanical properties of concrete obtained from the actual measurement results. Parameter *c* of the O-C40 concrete with lower strength decreases first and then increases; parameter *b* increases first and then decreases; and the curve steepens first and then slows down slightly, indicating that the ductility and residual strength of O-C40 concrete decreases first and then increases slightly. This is different from the law of gradual decrease of peak strength of O-C40 concrete. Parameter *b* of the O-C50 concrete also decreases first and then increases, indicating that the law of ductility change is similar to that of the O-C40 concrete. In Figure 6, the numerical change of parameter *a* of the model of [17] verified this phenomenon. In Figure 7c, the change of *R^2^_G_* also verified this phenomenon, which phenomenon can be seen in concrete with serious deterioration. Figure 7c shows the freeze–thaw data of another article: Through the quantitative analysis of the new model and the model of [17], it is found that the ductile deformation capacity of concrete decreases first and then increases slightly, which verifies the finding in this paper. Therefore, if the deterioration of concrete is serious, the deformation and flexibility of it does not decrease with the deepening on the deterioration of concrete, but decreases first and then slightly increases.

The ductile deformation capacity, plastic deformation capacity, and residual strength of ordinary concrete decrease first and then increase, so there must be a moment that the ductility and plastic deformation capacity of concrete are minimum. Take O-C40 concrete as an example to study the relationship between its ductile deformation capacity and the number of freeze–thaw cycles; its uniaxial compression failure process is shown in Figure 8.

**Figure 9:**
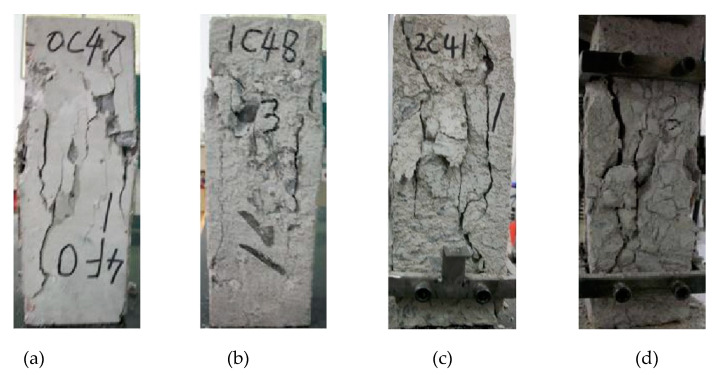
Failure mode of O-C40 concrete under uniaxial compression: (**a**) 0; (**b**) 50; (**c**) 100; (**d**) 150; 0, 50, 100, and 150 are numbers of freeze–thaw cycles.

The change rule of the fitting results (Table 2) indicates that the ductility and plastic deformation capacity of the O-C40 concrete is not completely reduced as the strength of the concrete decreases. As shown in Figure 9a,b, this is due to the high compactness of concrete in the early stage of freezing and thawing. At this time, the strength and toughness of concrete are the best. In the process of uniaxial compression of concrete, the higher compactness and integrity support the higher ductility and plastic deformation ability of concrete [33,34]. In the late stage of freezing and thawing, the O-C40 concrete has been damaged many times by freezing–thawing, the internal structure of concrete is deteriorated seriously, and the compactness and strength of concrete are low. As shown in Figure 9, the porosity and the degree of osteoporosis of concrete increase, and many micro-cracks appear. As the loading process progresses, the micro-cracks and loose parts of the concrete are closed by compression, showing a phenomenon of being compacted [27]; under small stress, there will be large deformation. Therefore, the plastic deformation starts to accelerate, and the concrete breaks and produces many small cracks when the loading force exceeds its bearing capacity; although the concrete has been damaged at this time, due to its low brittleness, it will not break into several large blocks in an instant. However, the internal small crack gradually expands and penetrates, eventually forming a large through crack; the failure process of concrete is not rapid. As shown in Figure 4, we can see the residual strength of the concrete after freeze–thaw cycle 150 times is higher than that of freeze–thaw 100 times. Therefore, the concrete still has certain ductility and plastic deformation ability in the later stage of freeze–thaw; in the middle of freeze–thaw, the porosity of concrete has increased, so its compactness and strength are lower than that of concrete in the initial stage of the freezing-and-thawing cycle, but higher than that of concrete in the later stage of freezing-and-thawing cycle. Due to the reduction of concrete strength, the toughness at this time is not enough to support sufficient ductility and plastic deformation of the concrete; also, because the concrete interior is not “loose” enough, the ductility and plastic deformation capacity of concrete are lower than that of late freeze–thaw. The brittle fracture of the concrete suddenly occurred during compression, forming multiple large cracks and breaking into several larger blocks, as shown in Figure 9c; its ductility and plastic deformation capacity decreased rapidly with the fracture of the concrete specimens. At this time, the ductility and plastic deformation ability of concrete are the weakest, the residual strength is the lowest, and the failure process is the fastest. Therefore, there is a moment when the ductility and plastic deformation capacity of the concrete are the smallest during the degradation process. This paper names this moment as the minimum moment of ductile deformation capacity of concrete (MDC).

In order to obtain the stress–strain curve of concrete in any deterioration period, and then to predict and study the mechanical properties of concrete, and also to explore the corresponding freeze-and-thaw times of the MDC, the relationship among the peak stress, the peak strain, parameters *c* in the ascending curve section, and parameter *b* in the descending curve section were established. Moreover, the number of freeze–thaw cycles of O-C40 concrete, O-C50 concrete, AE-C40 concrete, and AE-C50 concrete specimens under the action of freeze–thaw cycle were established by using the least square method, as shown in Equations (20)–(23):

(20) $$\{\begin{array}{l} f_{pr}=40.21-0.55043N+0.00573N^{2}-2.11067\times 10^{-5}N^{3}\\ \varepsilon_{pr}=0.00195+6.39733\times 10^{-5}N+4.296\times 10^{-7}N^{2}-2.72533\times 10^{-9}N^{3}\\ c=-0.68985-0.04235N-7.176\times 10^{-6}N^{2}+1.18004\times 10^{-6}N^{3}\\ b=0.78083-0.12899N+0.00451N^{2}-2.22899\times 10^{-5}N^{3} \end{array}$$

(21) $$\{\begin{array}{l} f_{pr}=52.87-0.4682N+0.00369N^{2}-1.148\times 10^{-5}N^{3}\\ \varepsilon_{pr}=0.00212+4.63667\times 10^{-5}N-2.82\times 10^{-7}N^{2}+1.41333\times 10^{-9}N^{3}\\ c=-0.68985-0.04235N-7.176\times 10^{-6}N^{2}+1.18004\times 10^{-6}N^{3}\\ b=1.13144-0.01004N+0.00118N^{2}-6.54031\times 10^{-6}N^{3} \end{array}$$

(22) $$\{\begin{array}{l} f_{pr}=45.36-0.00685N-1.88917\times 10^{-4}N^{2}+1.145\times 10^{-6}N^{3}-1.90833\times 10^{-9}N^{4}\\ \varepsilon_{pr}=0.00201+9.375\times 10^{-6}N-1.06375\times 10^{-7}N^{2}+4.525\times 10^{-10}N^{3}-5.625\times 10^{-13}N^{4}\\ c=-0.18862+0.00108N-2.71543\times 10^{-5}N^{2}+3.75995\times 10^{-8}N^{3}\\ b=2.22193+0.00266N+1.18126\times 10^{-5}N^{2}-2.49818\times 10^{-8}N^{3} \end{array}$$

(23) $$\{\begin{array}{l} f_{pr}=59.5-0.08837N+1.88\times 10^{-3}N^{2}-5.2225\times 10^{-6}N^{3}+6.62083\times 10^{-9}N^{4}\\ \varepsilon_{pr}=0.0017+7.4521\times 10^{-6}N-7.74979\times 10^{-8}N^{2}+3.1404\times 10^{-10}N^{3}-3.857\times 10^{-13}N^{4}\\ c=0.03453-0.00792N+3.67886\times 10^{-5}N^{2}-5.86856\times 10^{-8}N^{3}\\ b=2.9101+4.50523\times 10^{-4}N+1.78976\times 10^{-6}N^{2}-3.43367\times 10^{-9}N^{3} \end{array}$$

In Equations (20)–(23), *f_pr_* is the peak stress of concrete; *ε_pr_* is the peak strain of concrete; *b* and *c* are model parameters; and *N* is the number of freezing and thawing.

**Figure 10:**
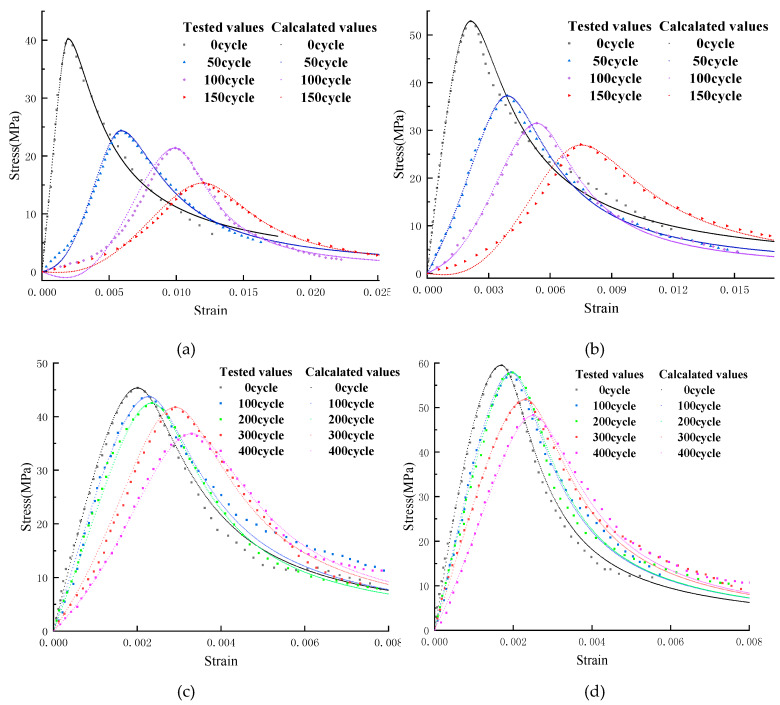
Comparison between the predicted stress–strain curve of freeze–thaw concrete and the measured one: (**a**) O-C40 concrete; (**b**) O-C40 concrete; (**c**) AE-C40 concrete; and (**d**) AE-C40 concrete.

According to Equations (20)–(23), the constitutive curve of freeze–thaw concrete in any deterioration period can be predicted. Figure 10 shows the comparison between the predicted result and the measured result. 

It can be seen from Figure 10 that the predicted curve has a high integration degree with the measured result, indicating that this method can be used to predict the geometric shape of the concrete constitutive curve for any number of freeze–thaw cycles, and further study the changes of mechanical properties of concrete such as peak stress, peak strain, ductile deformation ability, and residual strength.

According to Equation (20), the minimum value of the parameter *c* of O-C40 concrete is −3.865 and the corresponding number of freeze–thaw cycles is 111.42, the maximum value of the parameter *b* is 11.735 and the corresponding number of freeze–thaw cycles is 118.63, indicating that the MDC of the ordinary C40 concrete is about 111–118 times of freezing and thawing. According to Equation (21), the maximum value of the parameter *b* is 5.636, and the corresponding number of freeze–thaw cycles is 115.86, indicating that the MDC of the O-C50 concrete is about 116 times of freezing and thawing. 

In the study of this paper, the MDC only appears in ordinary concrete, but this phenomenon does not occur in air entraining concrete. The reason is that the ordinary concrete deteriorates faster; the compressive strength of O-C40 concrete and O-C50 concrete suffering 100 cycles of freezing and thawing decreased by 0.48 and 0.40, respectively, compared with that of unfreezing–thawing concrete, while that of AE-C40 concrete and AE-C50 concrete suffering 400 cycles of freezing and thawing decreased by 0.19 and 0.17, respectively, compared with that of unfreezing–thawing concrete. Therefore, the MDC usually occurs when the sample is damaged seriously, at this moment, the ductile deformation capacity of concrete is the weakest, the damage process is the fastest, and the concrete instantly break into several larger test blocks. Therefore, in practical engineering, we should not only pay attention to the change of concrete strength, but also the MDC, because once the concrete is destroyed when its ductility is the weakest, the damage speed of concrete is difficult to control and the loss is immeasurable. At present, the MDC can only be obtained by using the computing method in this paper. In the future, the research of the construction curve should pay attention to this aspect of the MDC, and it is also important to develop a more accurate forecasting methods for the MDC.

It can also be found in Figure 3 that the mass loss of concrete has not decreased to 5% and the relative dynamic elastic modulus has not decreased to 60% during the number of freeze–thaw cycles 100–150, but the MDC arrived, and the compressive strength of O-C40 concrete and O-C50 concrete also decreased by more than 0.48 and more than 0.40, respectively. It is obvious that during this period the compression concrete is prone to brittle fracture. Therefore, the evaluation of the frost resistance of concrete under low temperature or freeze–thaw environment cannot only refer to the changes of physical macroscopic index, and also should include the research of ductility deformation ability and mechanical index of concrete, in order to meet the requirements of safety assessment. 

## 5. Conclusions

In this paper, a new constitutive model of concrete under uniaxial compression was proposed, and its fitting characteristics and advantages were compared with traditional constitutive models. The applicability of the new model to the uniaxial compressive stress–strain curve of concrete under various degradation forms was verified, and the new model was used to quantitatively analyze and predict the stress–strain curve of concrete in different degradation periods. From the research in this paper, the following conclusions can be obtained:

(1) Compared with traditional constitutive models, the new model is simple in form and has few parameters; the parameter value can reflect the ductility and deformation of concrete. The fitting curve of the new model has the highest fitting degree with the measured stress–strain curve of concrete, and the goodness of fit (*R*^2^) is also the largest. The predicted curve of concrete obtained by the new model coincide with the measured constitutive curve.

(2) The new model is suitable for fitting the stress–strain curve of concrete under uniaxial compression under various deteriorating forms. For concrete with good mechanical properties, the fitting results of the new model are almost seamless with the measured results. For concrete with poor mechanical properties, the fitting results of the new model can still maintain a high degree of the goodness of fit with the measured results, and the fitting results are basically free of stress less than zero. Therefore, the fitting results of the new model are more accurate.

(3) The quantitative analysis results of the measured curve show that the ductile deformation capacity of concrete does not decrease with the deepening on the deterioration of concrete, but decreases first and then increase slightly. This phenomenon is often seen in concrete with serious deterioration, and the conclusion is also obtained by using the model of [17] to quantitatively analyze the measured curve.

(4) By collating the quantitative analysis results of the measured constitutive curve, it can be concluded that there is the MDC in the process of concrete deterioration. The MDC of O-C40 concrete is about 114 freeze–thaw cycles, and the MDC of O-C50 concrete is about 116 freeze–thaw cycles.

## Figures and Tables

**Table 1:** Concrete mix proportion (material consumption per cubic meter).

Grouping	Design Strength of Concrete	W/C Ratio	Water (kg)	Cement (kg)	Sand (kg)	Stone (kg)	W-R Agent (kg)	A-E Agent (kg)
1	O-C40	0.49	195	398	615	1192	-	-
2	O-C50	0.40	195	488	533	1184	-	-
3	AE-C40	0.42	160	380	622	1225	4.1	0.567
4	AE-C50	0.34	155	455	570	1265	6.5	2.000

**Table 2:** The parameter fitting results of the new model.

Number of Freeze–Thaw Cycles	O-C40		O-C50		Number of Freeze–Thaw Cycles	AE-C40		AE-C50	
	c	b	c	b		c	b	c	b
0	−0.69	0.78	−0.02	1.13	0	−0.19	2.24	0.04	2.91
50	−2.67	2.83	−1.23	2.76	100	−0.45	2.61	−0.44	2.98
100	−3.82	10.74	−2.26	5.37	200	−0.55	3.05	−0.56	3.05
150	−3.22	7.78	−3.27	4.06	300	−1.43	3.45	−0.60	3.11
—	—	—	—	—	400	−1.66	3.61	−1.01	3.16
